# “Losing the Brakes”—Suppressed Inhibitors Triggering Uncontrolled *Wnt*/*ß-Catenin* Signaling May Provide a Potential Therapeutic Target in Elderly Acute Myeloid Leukemia

**DOI:** 10.3390/cimb45010040

**Published:** 2023-01-09

**Authors:** Ghaleb Elyamany, Hassan Rizwan, Ariz Akhter, Mansour S. Aljabry, Sultan Alotaibi, Mohammad A. Hameed Albalawi, Meer-Taher Shabani-Rad, Tariq Mahmood Roshan, Adnan Mansoor

**Affiliations:** 1 Department of Central Military Laboratory and Blood Bank and Adult Clinical Hematology & Hematopoietic Stem Cell Transplantation, Prince Sultan Military Medical City, Riyadh 12233, Saudi Arabia; 2 Department of Pathology & Laboratory Medicine, University of Calgary/Alberta Precision Laboratories (APL), Calgary, AB T2N 1N4, Canada; 3 Department of Pathology, College of Medicine, King Saud University, Riyadh 12372, Saudi Arabia; 4 Department of Internal Medicine, College of Medicine, Taibah University, Medina 42353, Saudi Arabia

**Keywords:** acute myeloid leukemia, gene expression, *Wnt/β-catenin*, *Wnt/β-catenin* inhibitors, targeted therapy

## Abstract

Dysregulated *Wnt*/*β-catenin* signal transduction is implicated in initiation, propagation, and poor prognosis in AML. Epigenetic inactivation is central to *Wnt*/*β-catenin* hyperactivity, and *Wnt*/*β-catenin* inhibitors are being investigated as targeted therapy. Dysregulated *Wnt*/*β-catenin* signaling has also been linked to accelerated aging. Since AML is a disease of old age (>60 yrs), we hypothesized age-related differential activity of *Wnt*/*β-catenin* signaling in AML patients. We probed *Wnt*/*β-catenin* expression in a series of AML in the elderly (>60 yrs) and compared it to a cohort of pediatric AML (<18 yrs). RNA from diagnostic bone marrow biopsies (*n* = 101) were evaluated for key *Wnt*/*β-catenin* molecule expression utilizing the NanoString platform. Differential expression of significance was defined as >2.5-fold difference (*p* < 0.01). A total of 36 pediatric AML (<18 yrs) and 36 elderly AML (>60 yrs) were identified in this cohort. Normal bone marrows (*n* = 10) were employed as controls. *Wnt*/*β-catenin* target genes (MYC, MYB, and RUNX1) showed upregulation, while *Wnt*/*β-catenin* inhibitors (CXXR, DKK1-4, SFRP1-4, SOST, and WIFI) were suppressed in elderly AML compared to pediatric AML and controls. Our data denote that suppressed inhibitor expression (through mutation or hypermethylation) is an additional contributing factor in *Wnt*/*β-catenin* hyperactivity in elderly AML, thus supporting *Wnt*/*β-catenin* inhibitors as potential targeted therapy.

## 1. Introduction

Acute myeloid leukemia (AML) is a malignant neoplasm of myeloid stem cells (MSCs) [1]. The pathogenesis is defined by genetic aberrations resulting in disrupted balance between expression of proto-oncogenes and the controlling mechanisms [2]. Hence, genetic heterogeneity defines the basis for clinical risk stratification [3]. The disease is prevalent among older patients (>60 yrs) who exhibit high (fivefold) mortality compared with younger patients [4]. The conventional and emerging therapeutic regimens, with or without bone marrow transplant, are providing efficacious clinical outcome in younger patients [5]. However, such therapeutic interventions pose limited utility in older AML patients due to high therapeutic toxicity and accompanying comorbidities. Hence, palliative therapy remains the only option among more than half of the older AML patients [6,7]. In recent years, AML in older patients has emerged as a distinct and defined entity. This approach is improving insight into AML biology, steering the development of appropriate targeted therapies, thus minimizing toxicities [7]. The characterization of detailed disease biology in this distinct group is essential for the identification of critical and targetable pathways to devise and expand novel therapeutic regimens for older AML patients [4,8].

Signal transduction pathways are crucial cellular processes that modulate genetic expression for physiological functions to maintain homeostasis. However, aging is linked with aberrant function of multiple signaling pathways and other host of factors that maintain cellular health [9]. Wingless-Int (*Wnt*)/*β-catenin* pathways are a group of signal transduction pathways that carefully coordinate critical cellular functions such as cell proliferation, differentiation, and migration. These effects are executed through three distinct cascades, namely, canonical and two noncanonical pathways [10]. Extracellular ligands induce signaling by binding Wnt receptors on the plasma membrane and recruiting co-receptors, such as the Frizzled receptor and lipoprotein-receptor-related receptors (LRPs) in the canonical cascade. The non-canonical Wnt signaling regulates cytoskeletal dynamics and directional cell movement through Frizzled or ROR receptors to transduce Wnt/planar cell polarity (PCP) and other Wnt/receptor signaling cascades [11]. AML pathogenesis is influenced by *Wnt*/*β-catenin* hyperactivity through epigenetic dysregulation, resulting in an imbalance in regulatory molecules and upregulating activity of Wnt intermediaries [12]. These events rescue the main effector β-catenin in the canonical Wnt pathway from proteolysis and permit it to translocate to the nucleus for expressing genes that coordinate cellular proliferation [13]. Gene expression profile (GEP) has identified WNT effectors that are differentially expressed in AML patients, while targeted inhibition of *Wnt*/*β-catenin* signaling provides therapeutic options in AML pts [14].

*Wnt*/*β-catenin* inhibitors are a group of molecules that attenuate signal transduction pathways by saturating *Wnt*/*β-catenin* receptors and degrading cytosolic β-catenin [15]. Recent investigations have demonstrated how endogenous *Wnt*/*β-catenin* inhibitors as well as *Wnt*/*β-catenin*-targeted therapies interfered with AML progression in cell lines, yet none explored whether they could be used for reducing the progression of AML [16]. The effectiveness of *Wnt*/*β-catenin*-targeted therapies have also been observed in colorectal cancer [17]; however, the extent to which *Wnt*/*β-catenin* intermediaries pertain to AML pathogenesis in elderly patients remains unknown.

In this study, we conducted a comparative analysis of *Wnt*/*β-catenin* activity via mRNA expression in diagnostic bone marrow biopsy samples of older AML patients while employing normal bone marrow and pediatric AML patient samples as controls. Our results authenticate *Wnt*/*β-catenin* overexpression in AML patients, as previously reported [18]. Importantly, we noted that *Wnt*/*β-catenin* inhibitor suppression contributes to *Wnt*/*β-catenin* hyperactivity. We believe these data could provide bases for future insights into the molecular mechanisms of *Wnt*/*β-catenin* overexpression for developing *Wnt*/*β-catenin*-related targeted therapies suited towards older AML patients.

## 2. Materials and Methods

### 2.1. Patients and Samples

This retrospective study utilized formalin-fixed, paraffin-embedded (FFPE) diagnostic bone marrow (BM) biopsy samples (*n* = 36) from older AML patients (>60 yrs) (2011–2015). A cohort of pediatric AML (<18 yrs) (*n* = 36) and age-matched (>60 yrs) normal bone marrow samples (*n* = 10) were used as controls. The normal control group (*n* = 10) comprised lymphoma staging bone marrow in patients >60 years, who were negative for lymphoma or any other pathology. Diagnosis and classification were reviewed (AM, GEY, MTSR) according to the 2016 WHO classification system [19]. The tissue samples were harvested through microdissection at selective sites with maximum tumor cells, avoiding the contamination of normal cellular elements. The sample size was deemed adequate (http://bioinformatics.mdanderson.org/Microarray/SampleSize, accessed on 17 July 2022). FLT3 (ITD and/or TDK) and NPM1 mutation results, where available, were recorded from clinical files. We employed standard criteria for differential expression (2.5-fold change; *p* < 0.01 and false discovery rate (q value) of <0.05). The institutional ethics committee approved this study (HREBA.CC-16-0771-MODI dated 14 June 2022).

### 2.2. RNA Extraction and NanoString nCounter Assay

RNA was extracted using the Ambion Kit (ThermoFisher Scientific, Waltham, MA, USA) utilizing multiple cores (1 mm) harvested off areas with maximum tumor concentration. The RNA concentration was quantified using a Nanodrop UV-VIS spectrophotometer (Nanodrop Technologies, Wilmington, DE, USA) and integrity was assessed using a Bio-analyzer 2100 and RNA Nano Chip assay (Agilent Technologies, Wilmington, DE, USA). Total RNA samples were processed according to the manufacturer’s protocol for the nCounter gene expression PAN-cancer pathway code set containing 770 key genes related to major pathways in cancer biology (NanoString, Seattle, WA, USA). Briefly, using nCounterTM technology, mRNA expression analysis was conducted for each sample using specific probes. Probes were hybridized to 300 ng of total RNA for 20 h at 65 °C and applied to the nCounterTM Prep Station for automated removal of excess probe and immobilization of probe–transcript complexes on a streptavidin-coated cartridge. Data were collected using the nCounterTM Digital Analyzer by counting the individual barcodes. mRNA analysis and normalization of the raw data were conducted using nSolver Analysis Software v3.0 (NanoString Technologies). mRNA raw counts were normalized to internal levels of 40 reference genes. Normalized data were log2-transformed and then used as input for further analysis.

### 2.3. Statistical Analysis

We used nSolver software v3.0 (NanoString Technologies) for the normalization of raw counts for various genes as determined by the NanoString nSolver platform. SPSS software v24.0 (IBM, Armonk, NY, USA) was utilized for other statistical evaluations. Hierarchical clustering and principal component analyses were performed employing Qlucore Omics Explorer v3.2 (Lund, Sweden). Results with fold change ≥2.0 and *p*-value < 0.05 were considered significant.

## 3. Results

**Figure 1:**
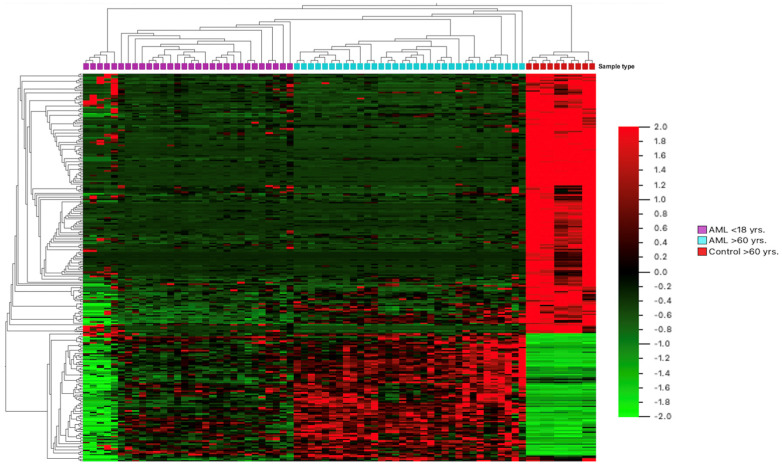
The hierarchical clustering analysis of the differential gene expression pattern between normal controls, elderly AML patients, and pediatric AML patients.

**Figure 2:**
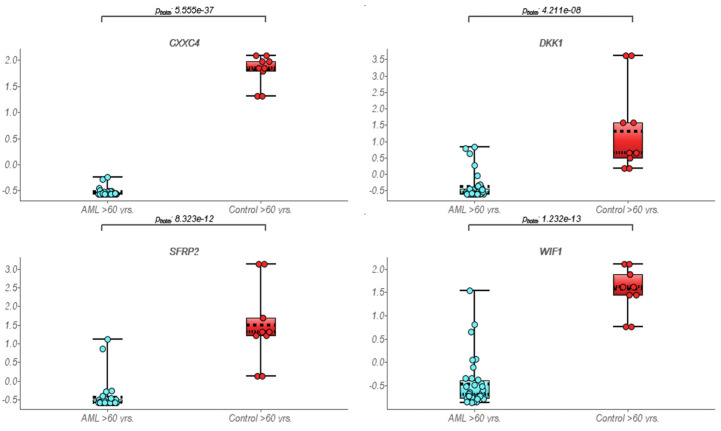
Box plot representing suppressed expression of selective *Wnt*/*β-catenin* inhibitor genes among elderly AML samples compared to normal controls.

**Figure 3:**
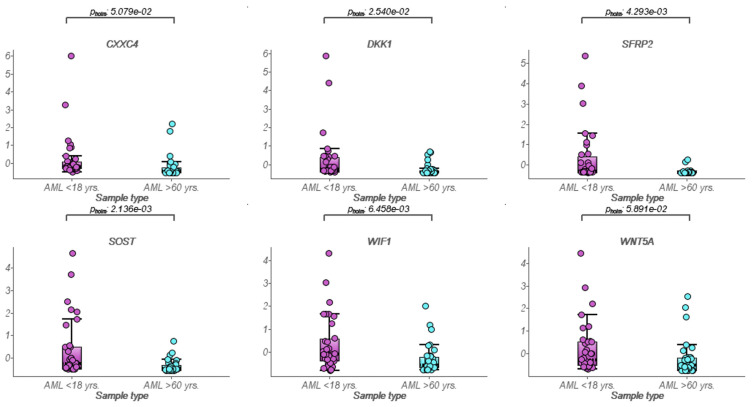
Box plot representing suppressed expression of selective *Wnt*/*β-catenin* inhibitors among elderly AML compared to pediatric AML patients.

A total of 36 elderly AML patients (median age of 74 years; range 60–83 years; 20 men/16 women, M:F 1.25:1) were included. The median bone marrow blast count was 61% (range 46–90%). All patients were investigated on the basis of a standardized protocol. No significant correlation was observed between blast count and median counts for the expression of *Wnt*/*β-catenin* pathway molecules in this group (Person correlation, r (0.21); *p* = 0.31). A total of 12/36 (33%) patients were positive for both FLT3 and NPM1 mutations. The FLT3+/NPM1− mutation was noted in only 4/36 (11%), while 10/36 (28%) patients were positive for the NPM1 mutation only. In total, 10/36 (28%) were negative for both FLT3 and NPM1 mutations. No distinct differential expression was noted between FLT3 and/or NPM1 mutational status for *Wnt*/*β-catenin* molecules. The pediatric group comprised 36 pediatric AML patients (median age of 14 years; range 3 month–18 years; 22 male/14 female, M:F 1.57:1). The median bone marrow blast count in this cohort was 53% (range 42–98%). The median expression counts for *Wnt*/*β-catenin* pathway molecules did not relate to blast counts (Person correlation, r (0.17); *p* = 0.73). In this group, 08/36 (22%) patients were positive for both FLT3 and NPM1 mutations. The FLT3+/NPM1− mutation was noted in only 1/36 (3%), while 07/36 (19%) patients were positive for the NPM1 mutation only. A total of 11/36 (30%) were negative for both FLT3 and NPM1 mutations, while in 9/36 (25%) patients, the FLT3/NPM1 mutational status was unknown. GEP by hierarchical clustering revealed a distinct pattern in older AML as compared to pediatric AML or normal controls (Figure 1). We identified three distinct *Wnt*/*β-catenin* target genes, namely, MYC, MYB, and RUNX1, which showed upregulation in elderly AML compared to the pediatric AML group, thus confirming upregulated *Wnt*/*β-catenin* pathway molecules. There were 11 genes defined as inhibitors of *Wnt*/*β-catenin* pathway exhibiting low expression among the elderly AML cohort compared to pediatric AML patients (Table 1). We noted statistically significant lower expression of selective *Wnt*/*β-catenin* inhibitors in elderly AML compared to normal control samples as well as pediatric AML samples (Figure 2 and Figure 3).

## 4. Discussion

AML in elderly patients is a distinct disease due to its discrete biology and poor prognosis [7]. The conventional AML therapeutic options are often rendered less effective and more toxic in the long term due to several comorbidities [4,6]. Increased *Wnt*/*β-catenin* signaling contributes towards AML promotion by enhancing cellular growth through several mechanisms, including mutations and epigenetic variations of effectors that moderate these processes [12]. *Wnt*/*β-catenin* inhibitors by attenuating *Wnt*/*β-catenin* signaling are thought to coordinate tissue growth and specialization during embryonic development and tissue homeostasis [20], but their impact on the genomic biology of AML remains complex and evolving [21]. Although previous cell line experiments demonstrated the importance of *Wnt*/*β-catenin* signaling in various other cancers and physiological processes, the role of aging on *Wnt*/*β-catenin* inhibitor activity has not been investigated [17,22]. Hence, studying *Wnt*/*β-catenin* expression in detail could help in devising additional therapeutic options in elderly AML patients. To expand our understanding, we performed a comprehensive comparative GEP analysis in elderly AML against matched normal controls and compared it with pediatric AML patients. We captured holistic data related to tumor cells as well as the influence of the tumor microenvironment across several signal transduction pathways. Our study reported increased *Wnt*/*β-catenin* signaling in the elderly AML cohort compared to the pediatric AML cohort. We also ascertained that several *Wnt*/*β-catenin* inhibitors were significantly repressed amid elderly AML patients.

The mutational landscape in AML has a discrete impact on clinical progression, and the WHO classification system has defined distinct AML subtypes based on FLT3 and NPM1 mutations [19]. It is empirical to evaluate and correlate emerging insights in AML biology with these conventional prognostic makers. There are reports linking a positive or negative correlation between *Wnt*/*β-catenin* pathway expression and FLT3/NPM1 mutations in AML [23,24]. However, this pilot study failed to document any statistically significant association between *Wnt*/*β-catenin* pathway expression and FLT3/NPM1 mutational status either in the elderly AML or pediatric AML group. This may be attributed to the small sample size and there being several intricate molecules within a complex network. There was no further attempt made to collectively analyze FLT3/NPM1 mutation status within *Wnt*/*β-catenin* pathway molecules.

The secreted frizzled related family (SFRP) of *Wnt*/*β-catenin* inhibitors consists of a broad group of ligands released by a subset of cellular elements for mediating homeostasis and modeling the microenvironments of vascular tissues [25]. However, SFRPs have also been identified to exert onco-suppressive and oncogenic roles in different tissues and cancers [26,27]. We found SFRP2 was downregulated (>10-fold) the most out of these genes in the elderly AML samples. Although the methylation of its promoter is associated with AML progression, the molecular mechanisms by which SFRP2 prevents AML are unknown, apart from inhibiting extracellular matrix remodeling [28]. The influence of SFRP2’s downstream effector molecules, especially TP53, which is frequently dysfunctional in elderly AML, deserve further investigation since overexpression of SFRP2 in TP53-deficient osteoblasts is linked with osteosarcoma development [29]. Moreover, TP53 mutation confers tumor resistance among most elderly AML patients, worsening their clinical outcomes [30]. Like SRFP2, the methylation of SRFP1 promoters has been associated with AML development, yet the regulatory mechanism of its impact on *Wnt*/*β-catenin* signaling remains unknown [31]. SFRP1 has previously been established as the more prevalent biomarker in AML patients, whereas SFRP4 is rarely detected [32]. SFRP1 is also found to be epigenetically repressed alongside NPM1, a prominent prognostic factor of AML [32]. Hence, our data support the notion that epigenetic manipulation of SRFP family members could pose another option for inhibiting *Wnt*/*β-catenin* signaling for elderly AML patients to enhance remission.

Additionally, our study detected a distinct difference in the expression profile of Dikkop (DKK) family molecules between the two age groups within AML. These molecules mediate several signal transduction pathways for coordinating homeostasis [33]. Selective DKK ligands function as *Wnt*/*β-catenin* inhibitors and are associated in AML progression inferring oncogenic and tumor-suppressive properties depending on the tumor and its microenvironment [34]. In this respect, DKK1 is emerging as a molecule with multifaceted functions. The effect of DKK1 in progression of cancer both in in vitro and in vivo models is well established [35]. In AML, DKK1 is released by cancer cells to alter the stromal microenvironment for propagating the disease while hindering normal hematopoietic stem cell (HSC) activity. Excessive DKK1 production could indirectly establish a tumor niche, resulting in AML progression, while suppression of DKK1 by exogenous agents delayed AML progression and prolonged survival in animal models [36]. This effect is independent of DKK1 function as a *Wnt*/*β-catenin* inhibitor. Furthermore, DKK1 is also secreted by malignant bone marrow stromal cells for inhibiting the adaptive immune response and expansion of myeloid-derived suppressor cells (MDSC), hence permitting immune evasion [37]. In this context, immunotherapy against DKK1 is being considered as a therapeutic tool and may have a benefit in being explored as a treatment modality in elderly AML [38].

We also found DKK2 and DKK4 were downregulated in elderly patients, but to a lesser degree than the other *Wnt*/*β-catenin* inhibitors. They are implicated in the neoplastic processes with or without DKK1 expression, indicating they can mediate an independent impact on AML progression [39]. However, their functions may vary with factors intrinsic to the tissue microenvironment. Coincidentally, we also found that IDAX (the product of CXXC4) expression was also downregulated in elderly AML patients. CXXC4 is implicated as having an onco-suppressor function in solid tumors [40]. Although the role of CXXC4 is not currently well understood in AML, future investigations could explore it as another therapeutic target for addressing treatment options in elderly AML.

The insight into the molecular mechanisms leading to the downregulation of *Wnt*/*β-catenin* inhibitors in AML are sketchy. Hypermethylation, being the most rationale mechanism, has been reported in a subset (8% to 54%) of AML patients [41]. This implies additional and diverse mechanisms for low expression of *Wnt*/*β-catenin* inhibitors in AML. This is further supported by epigenetic influence through high expression of micro-RNA impacting *Wnt*/*β-catenin* inhibitor (DKK3) as reported in adult B-cell lymphoblastic leukemia [42]. These additional mechanisms may be influencing the efficacy of the hypomethylating agents in elderly AML, which is at best moderate [43]. In solid organ tumors, this subject is reviewed in detail, and several unspecified mechanisms have been suggested [44]. Hence, additional approaches including combination therapies such as Venetoclax and other investigational agents are being under extensive evaluation [45]. Since supplemental pharmacotherapies with exogenous *Wnt*/*b-catenin* inhibitors are providing benefits in solid cancers, such agents may have therapeutic potential in elderly AML as well [46]. It will be prudent to explicitly state that observations presented in this report should be inferred in the context of the limitations of this pilot study. These extrapolations require validation through future comprehensive studies to link RNA expression levels with proteomics data. However, we can reflect some confidence in our findings through indirect evidence from the current literature. Several studies have reported good correlation between *Wnt*/*β-catenin* transcripts with protein expression, either through immunohistochemistry or Western blotting in solid cancers and other pathological processes [47,48,49,50].

## 5. Conclusions

Taken together, our study demonstrates that elevated *Wnt*/*β-catenin* signaling is implicated in clinical biopsies of elderly AML at diagnosis. Some effector molecules are repressed to a greater extent than others, suggesting the disease-founding mechanisms related to *Wnt*/*β-catenin* signaling are distinct in elderly AML and are independent of the normal aging process. Furthermore, the GEPs indicate re-routing of other signal transduction pathways, emphasizing the inclusion of expression profile into the genetic analysis to explore biology and prospects of target discovery. The restoration of suppressed *Wnt*/*β-catenin* inhibitors may offer a novel and less toxic strategy in the management of elderly AML, although such an approach mandates harnessing DKK1 suppression of host immunity.

## Figures and Tables

**Table 1:** Distinct lower mRNA expression of the selected *Wnt*/*β-catenin* pathway inhibitors in elderly AML patients compared to pediatric AML patients.

Genes	*p*-Value	q-Value	Fold Change
*AXIN2*	1.38 × 10^−5^	4.98 × 10^−5^	−3.1
*CXXC4*	1.74 × 10^−7^	1.08 × 10^−6^	−3.5
*DKK1*	8.87 × 10^−7^	4.96 × 10^−6^	−4.7
*DKK2*	0.008930	0.012775	−2.5
*DKK4*	0.000645	0.001336	−3.1
*SFRP1*	2.82 × 10^−6^	1.27 × 10^−5^	−4.3
*SFRP2*	7.48 × 10^−10^	1.04 × 10^−8^	−9.2
*SFRP4*	2.52 × 10^−5^	8.32 × 10^−5^	−4.5
*SOST*	6.67 × 10^−6^	2.56 × 10^−5^	−4.5
*WIF1*	0.005997	0.008683	−2.6
*WNT5A*	0.000274	0.000636	−2.5

## Data Availability

Data sharing not applicable. No new data were created or analyzed in this study. Data sharing is not applicable to this article.
